# Association of *MAPT* haplotype‐tagging polymorphisms with cerebrospinal fluid biomarkers of Alzheimer's disease: A preliminary study in a Croatian cohort

**DOI:** 10.1002/brb3.1128

**Published:** 2018-10-17

**Authors:** Mirjana Babić Leko, Nanet Willumsen, Matea Nikolac Perković, Nataša Klepac, Fran Borovečki, Patrick R. Hof, Zdenko Sonicki, Nela Pivac, Rohan de Silva, Goran Šimić

**Affiliations:** ^1^ Department for Neuroscience Croatian Institute for Brain Research, University of Zagreb Medical School Zagreb Croatia; ^2^ Reta Lila Weston Institute, UCL Institute of Neurology London UK; ^3^ Department of Molecular Neuroscience UCL Institute of Neurology London UK; ^4^ Ruđer Bošković Institute Division of Molecular Medicine Zagreb Croatia; ^5^ Department for Functional Genomics, Center for Translational and Clinical Research University of Zagreb Medical School, University Hospital Center Zagreb Zagreb Croatia; ^6^ Fishberg Department of Neuroscience Friedman Brain Institute and Ronald M. Loeb Center for Alzheimer's Disease, Icahn School of Medicine at Mount Sinai New York New York; ^7^ Andrija Štampar School of Public Health University of Zagreb School of Medicine Zagreb Croatia

**Keywords:** Alzheimer's disease, biomarkers, cerebrospinal fluid, genetic predisposition to disease, single‐nucleotide polymorphism, tau proteins

## Abstract

**Introduction:**

Alzheimer's disease (AD) is the world leading cause of dementia. Early detection of AD is essential for faster and more efficacious usage of therapeutics and preventive measures. Even though it is well known that one ε4 allele of apolipoprotein E gene increases the risk for sporadic AD five times, and that two ε4 alleles increase the risk 20 times, reliable genetic markers for AD are not yet available. Previous studies have shown that microtubule‐associated protein tau (*MAPT*) gene polymorphisms could be associated with increased risk for AD.

**Methods:**

The present study included 113 AD patients and 53 patients with mild cognitive impairment (MCI), as well as nine healthy controls (HC) and 53 patients with other primary causes of dementia. The study assessed whether six *MAPT* haplotype‐tagging polymorphisms (rs1467967, rs242557, rs3785883, rs2471738, del–In9, and rs7521) and *MAPT* haplotypes are associated with AD pathology, as measured by cerebrospinal fluid (CSF) AD biomarkers amyloid β_1–42_ (Aβ_1–42_), total tau (t‐tau), tau phosphorylated at epitopes 181 (p‐tau_181_), 199 (p‐tau_199_), and 231 (p‐tau_231_), and visinin‐like protein 1 (VILIP‐1).

**Results:**

Significant increases in t‐tau and p‐tau CSF levels were found in patients with AG and AA *MAPT* rs1467967 genotype, CC *MAPT* rs2471738 genotype and in patients with H1H2 or H2H2 *MAPT* haplotype.

**Conclusions:**

These results indicate that *MAPT* haplotype‐tagging polymorphisms and *MAPT* haplotypes should be further tested as potential genetic biomarkers of AD.

## 1 INTRODUCTION

Alzheimer’s disease (AD), the most common primary cause of dementia, is a complex disease with poorly understood etiology. The characteristic amyloid plaques and neurofibrillary changes seen in AD brains are frequently observed in other neurodegenerative diseases. As a consequence, many AD cases are misdiagnosed. One such autopsy‐confirmed series showed sensitivity of AD diagnosis to range from 70.9% to 87.3%, while specificity ranged from 44.3% to 70.8% (with controls as reference groups; Beach, Monsell, Phillips, & Kukull, 2012; Gay, Taylor, Hohl, Tolnay, & Staehelin, 2008; Joachim, Morris, & Selkoe, 1988). This creates substantial difficulties in interpretations of results obtained by different studies that include patients with probable AD. One possibility to avoid this problem would be to use intermediate quantitative traits (endophenotypes) rather than clinical diagnoses (case–control studies) as indices of AD pathology. Endophenotypes are any biomarkers that signal the presence of AD pathology. For example, for this study, we used core cerebrospinal fluid (CSF) biomarkers of AD, such as amyloid β_1–42_ (Aβ_1–42_), total tau (t‐tau), and tau phosphorylated at epitope 181 (p‐tau_181_), and potential CSF biomarkers, such as tau phosphorylated at epitopes 199 (p‐tau_199_), and 231 (p‐tau_231_), and visinin‐like protein 1 (VILIP‐1) as endophenotypes. Aβ_1–42_ indicates the presence of senile plaques in the brain (Grimmer et al., 2009), t‐tau and VILIP‐1 are markers of neurodegeneration (Babić et al., 2014; Babić Leko, Borovečki, Dejanović, Hof, & Šimić, 2016), while p‐tau_181_, p‐tau_199_, and p‐tau_231_ reflect the presence of neurofibrillary tangles in the brain (Bürger et al., 2006). CSF core biomarkers (Aβ_1–42_, t‐tau, and p‐tau_181_) were previously used in genome‐wide association studies (GWAS) as endophenotypes for detection of AD risk genes (Cruchaga et al., 2013; Kim et al., 2011). In this study, we used these biomarkers to assess whether certain variants of microtubule‐associated protein tau (*MAPT*) gene were associated to their pathological levels in CSF. Although AD is not caused by mutations in the *MAPT* gene, previous studies demonstrated that CSF biomarkers of AD differ among patients with different *MAPT* genotypes (Compta et al., 2011; Kauwe et al., 2008). Besides comparing the levels of CSF biomarkers Aβ_1–42_, t‐tau, p‐tau_181_, p‐tau_199_, p‐tau_231_, and VILIP‐1 among patients with six different *MAPT* genotypes, we also analyzed the distribution of *MAPT* H1 and H2 haplotypes and their subhaplotypes in a Croatian patient cohort.

The majority of AD patients are late‐onset sporadic cases, whose heritability for the disease has been estimated to be as high as 58%–79% (Gatz et al., 2006). In addition to apolipoprotein E gene (*APOE*), more than 20 common loci have been associated with risk for sporadic AD, age at onset, and progression of cognitive decline, but reported genome‐wide significant loci do not account for all the estimated heritability and provide little information about underlying biological mechanisms (Šimić et al., 2016a, 2016b). Therefore, genetic studies like the present one, using intermediate quantitative traits (endophenotypes) such as biomarkers, greatly benefit from increased statistical power to identify variants that may not pass the stringent multiple test corrections in case–control studies.

## 2 MATERIALS AND METHODS

### 2.1 Subjects

The study was conducted at Clinical Hospital Center Zagreb from August 2011 until June 2016. All patients signed informed consent form for lumbar puncture and for participation in this study. The consent forms were explained in details to the patients and their guardians/caregivers. The study included 116 female (F) and 112 male (M) subjects: 113 AD patients (60F/53M) and 53 mild cognitive impairment (MCI) patients (27F/26M), nine healthy controls (HC, 6F/3M) and 53 patients with other causes of dementia (22 with frontotemporal dementia [FTD, 11F/11M], 14 with vascular dementia [VaD, 6F/8M], seven with dementia with Lewy bodies [DLB, 2F/5M], four with nonspecific dementia [ND, 3F/1M], three with mixed dementia [AD+VaD, 3M], two with Parkinson’s disease [PD, 2 M], and one with corticobasal syndrome [CBS]), all recruited at the University Hospital Center, Zagreb (Table 1).

**Table 1:** Levels of CSF protein biomarkers and demographic data

	Aβ_1__–__42_ (pg/ml)		Total tau (pg/ml)		p‐tau_181_ (pg/ml)		p‐tau_199_ (pg/ml)		p‐tau_231_ (U/ml)		VILIP‐1 (pg/ml)		MMSE	Age
	Mean ± *SD* (number of patients)	Median (25–75th percentile)	Mean ± *SD* (number of patients)	Median (25–75th percentile)	Mean ± *SD* (number of patients)	Median (25–75th percentile)	Mean ± *SD* (number of patients)	Median (25–75th percentile)	Mean ± *SD* (number of patients)	Median (25–75th percentile)	Mean ± *SD* (number of patients)	Median (25–75th percentile)	Mean ± *SD* (number of patients)	Median (25–75th percentile)
AD	539.39 ± 298.92 (113)	505.51 (308–725.5)	507.45 ± 372.53 (113)	507.45 (246.5–652.83)	79.88 ± 47.96 (112)	70.94 (55.25–93.65)	4.37 ± 3.53 (113)	3.56 (1.72–6.19)	3.86 ± 5.52 (111)	1.74 (0.67–3.72)	137.12 ± 88.58 (111)	121.73 (57.57–194.87)	19.83 ± 4.85 (113)	73 (67–77)
MCI	723.44 ± 371.87 (53)	679 (398.02–1,023)	246.44 ± 158.00 (53)	210 (134.5–330.17)	57.64 ± 30.86 (51)	51.96 (36.37–69.08)	3.40 ± 2.35 (52)	2.92 (1.37–5.21)	1.82 ± 3.18 (51)	0.82 (0.36–1.94)	94.86 ± 78.11 (50)	70.12 (27–135.04)	25.09 ± 2.96 (53)	70 (59–74)
HC	860.33 ± 497.46 (9)	1,022 (265–1,315)	293.33 ± 346.14 (9)	228 (62–367)	48.54 ± 26.14 (9)	55.82 (17.22–70.18)	1.42 ± 0.96 (9)	1.42 (0.72–2.42)	1.29 ± 2.05 (9)	0.51 (1.67–1.70)	99.27 ± 70.36 (9)	109.25 (27–160.30)	27.43 ± 1.81 (9)	52 (43–62)
FTD	391.17 ± 175.17 (22)	381.5 (276.78–532.1)	502.02 ± 408.99 (22)	322.02 (201.45–773.57)	69.62 ± 37.89 (22)	71.44 (31.76–106.81)	5.92 ± 5.52 (22)	4.34 (2.31–7.29)	1.91 ± 2.34 (22)	1.05 (0.69–1.93)	104.21 ± 73.30 (22)	77.29 (39.95–174.94)	16.32 ± 5.31 (22)	61 (56–65)
VaD	502.91 ± 235.95 (14)	432.13 (364.25–714.7)	516.31 ± 325.75 (14)	409.53 (310.73–742.47)	72.68 ± 36.22 (13)	72.9 (45.59–111.18)	5.30 ± 5.51 (14)	2.89 (1.60–7.89)	1.44 ± 0.78 (13)	1.54 (0.79–2.06)	123.03 ± 78.30 (13)	105.64 (47.04–173.57)	23.50 ± 5.04 (14)	71 (63–77)
DLB	428.32 ± 259.78 (7)	296.13 (200.68–657.11)	126.41 ± 44.91 (7)	130.03 (81.09–153.00)	41.99 ± 28.84 (7)	34.24 (25.79–39.98)	2.48 ± 1.58 (7)	2.58 (0.89–3.67)	0.56 ± 0.18 (7)	0.57 (0.48–0.67)	47.80 ± 16.90 (7)	48.02 (29.22–64.23)	19.29 ± 3.90 (7)	70 (68–75)
AD + VaD	553.23 ± 37.17 (3)	535	579.67 ± 460.42 (3)	661	74.11 ± 28.57 (3)	89.49	2.84 ± 1.93 (3)	3.75	1.97 ± 1.80 (3)	2.12	108.03 ± 70.19 (3)	146.88	19.33 ± 4.04 (3)	78
ND	459.46 ± 194.13 (4)	465.31 (273.27–639.80)	230.68 ± 116.96 (4)	239.30 (118.96–333.78)	25.48 ± 8.22 (4)	26.49 (17.19–32.77)	1.66 ± 1.94 (4)	1.48 (0.00–3.50)	0.39 ± 0.27 (4)	0.42 (0.41–0.63)	57.26 ± 60.52 (4)	27 (27–117.78)	19.25 ± 5.32 (4)	66 (52–68)
PD	232.08 ± 139.89 (2)	282.08	30.5 ± 43.13 (2)	30.5	20.48 ± 16.33 (2)	20.48	1.26 ± 1.31 (2)	1.26	0.35 ± 0.31 (2)	0.35	27 (2)	27	30 (1)	78
CBS	917.56 (1)		578.28 (1)		84.3 (1)		1.66 (1)		0.81 (1)		347.62 (1)		27 (1)	51

Note

Aβ_1–42_: amyloid β_1–42_ protein; AD: Alzheimer's disease; AD + VaD: mixed dementia; CBS: corticobasal syndrome; DLB: dementia with Lewy bodies; FTD: frontotemporal dementia; HC: healthy control; MCI: mild cognitive impairment; ND: nonspecific dementia; p‐tau_181_: tau protein phosphorylated at threonine 181; p‐tau_231_: tau protein phosphorylated at threonine 231; p‐tau_199_: tau protein phosphorylated at serine 199; PD: Parkinson’s disease; *SD*: standard deviation; VaD: vascular dementia.

This research study had a diagnostic purpose exclusively and was not designed to evaluate any health‐related interventions or possible effects on health outcomes. Therefore, it was not registered as a clinical trial. All patients were neuropsychologically tested using the Mini‐Mental State Examination (MMSE), Montreal Cognitive Assessment (MoCA), and Alzheimer’s Disease Assessment Scale‐cognitive subscale (ADAS‐Cog), underwent neurological examination and complete blood tests including levels of vitamin B_12_, folic acid (B_9_), and thyroid function test, and had a negative serology for syphilis or Lyme’s disease. All procedures were approved by the Ethical Committee of the Clinical Hospital Center Zagreb and by the Central Ethical Committee of the University of Zagreb Medical School (case no. 380‐59/11‐500‐77/90, class 641‐01/11‐02), and were in accord with the Helsinki Declaration (World Medical Association, 2013).

For AD, we used diagnostic criteria of McKhann et al. (2011), while MCI was diagnosed according to Petersen et al. (1999) and Albert et al. (2011). For VaD, we used the National Institute for Neurological Disorders and Stroke—Association Internationale pour la Recherche et l'Enseignement en Neurosciences (NINCDS‐AIREN) criteria (Román et al., 1993), as well as the Hachinski Ischemic Score (HIS) (Hachinski et al., 1975). The most important clinical discriminative factors for VaD vs. AD are stepwise progression, prominent impairment of the executive functions, higher probability of VaD when HIS is >4, and focal neurological signs implying cortical or subcortical lesions (Desmond et al., 1999). Clinical criteria for FTD were based on consensus published by Neary and collaborators (Neary et al., 1998). Conditions overlapping AD are very difficult to study, but biomarkers that we have chosen are again superior in this respect in comparison to other approaches, such as correlation with clinical diagnosis.

### 2.2 Lumbar puncture and ELISA analysis of CSF

Cerebrospinal fluid was taken by lumbar puncture between intervertebral spaces L3/L4 or L4/L5. After centrifugation at 2,000 *g* for 10 min, CSF samples were aliquoted and stored in polypropylene tubes at −80°C. Levels of Aβ_1–42_, t‐tau, p‐tau_231_, p‐tau_199_, p‐tau_181_, and VILIP‐1 were determined using the following enzyme‐linked immunosorbent assays (ELISA): Aβ_1–42_ (Innotest β‐amyloid_1–42_, Fujirebio, Gent, Belgium), t‐tau (Innotest hTau Ag, Fujirebio), p‐tau_231_ (Tau [pT231] Phospho‐ELISA Kit, Human, Thermo Fisher Scientific, Waltham, MA), p‐tau_199_ (TAU [pS199] Phospho‐ELISA Kit, Human, Thermo Fisher Scientific), p‐tau_181_ (Innotest Phospho‐Tau (181P), Fujirebio), and VILIP‐1 (VILIP‐1 Human ELISA, BioVendor, Brno, Czech Republic), respectively. Each CSF sample was analyzed in duplicate. Concentrations ranges of each biomarker are listed in Table 1. Cutoff values of CSF biomarkers were determined by ROC (receiver operating characteristic) curve analysis.

### 2.3 DNA analysis of *MAPT* polymorphisms

Venous blood samples (4 ml) were collected into plastic syringes with 1 ml of acid citrate dextrose as an anticoagulant. Genomic DNA was extracted from peripheral blood using the salting‐out method (Miller, Dykes, & Polesky, 1988). *MAPT* gene polymorphisms (rs1467967, rs242557, rs3785883, rs2471738, del–In9, and rs7521) were determined using primers and probes purchased from Applied Biosystems as TaqMan® SNP Genotyping Assay by ABI Prism 7300 Real Time PCR System apparatus (Applied Biosystems, Foster city, CA). The del‐In9 deletion in *MAPT* Intron 9 defines the H1/H2 *MAPT* haplotype division caused by the inversion. H1 and H2 *MAPT* subhaplotypes were determined using haplotype tagging SNPs in following order: rs1467967, rs242557, rs3785883, rs2471738, del–In9, and rs7521 (Table 2).

**Table 2:** Number of H1 and H2 *MAPT* subhaplotypes in the present cohort

Subhaplotypes	htSNP alleles	Number of alleles	Percentage (%) of alleles
H1B	GGGCAA	92	19.83
H1c	AAGTAG	58	12.50
H1E	AGGCAA	43	9.27
H1D	AAGCAA	37	7.97
H1l	AGACAG	30	6.47
H1u	AAGCAG	17	3.66
H1h	AGACAA	12	2.59
H1i	GAGCAA	10	2.16
H1J	AGGCAG	10	2.16
H1m	GAGCAG	10	2.16
H1T	AGATAG	10	2.16
H1k	AAATAG	9	1.94
H1jj	GGGCAG	8	1.72
H1o	AAACAA	8	1.72
H1v	GGATAG	7	1.51
H1q	AAGTAA	6	1.29
H1g	GAACAA	5	1.08
H1x	GAATAG	5	1.08
H1y	GAACAG	5	1.08
H1F	GGACAA	4	0.86
H1p	GGGTAG	3	0.65
H1aa	GAGTAG	2	0.43
H1r	AGGTAG	1	0.22
H2A	AGGCGG	56	12.07
H2gg	AGACGG	12	2.59
H2ff	AAGCGG	2	0.43
H2kk	AGGCGA	1	0.22
H2w	GGGCGA	1	0.22

### 2.4 Experimental design and statistical analysis

All statistical analyses were performed in SPSS 19.0.1 (SPSS, Chicago, IL, USA) with the statistical significance level set at *α* = 0.05. The Kolmogorov–Smirnov test was used for assessing data distribution normality. Regardless of the results of the Kolmogorov–Smirnov test for normality, due to the small number of patients in certain groups, nonparametric tests (Kruskal–Wallis test and Mann–Whitney *U* test) were used for comparison of the biomarkers’ levels between groups of subjects with different *MAPT* genotypes and haplotypes. After Kruskal–Wallis test for pairwise comparisons of independent samples, we performed the *post hoc* analysis. Since this *post hoc* testing option in SPSS incorporates calculation of the corrected *p*‐value, there was no need for additional corrections due to multiple comparisons. Nevertheless, due to the relatively small sample size, we performed statistical analysis with and without outliers. Only one statistically significant difference was lost after exclusion of outliers. Only results of the statistical analysis without outliers are presented here.

### 2.5 Ethical approval

The present study was conducted according to the 6th revised Declaration of Helsinki (Edinburgh, 2000) and Good Clinical Practice Guidelines and was approved by the local ethics committee of the Clinical Hospital Centre Zagreb. All the patients signed informed consent form for lumbar puncture, genetic research and for participation in this study. The consent form was explained in details to the patients and their legal guardians/caregivers. All procedures were approved by the Central Ethical Committee of the University of Zagreb Medical School (case no. 380‐59/11‐500‐77/90, class 641‐01/11‐02, signed on 19 May 2011).

## 3 RESULTS

### 3.1 Comparison of protein CSF biomarkers between subjects with different *MAPT* genotypes

The concentrations of CSF protein biomarkers (Aβ_1–42_, t‐tau, p‐tau_181_, p‐tau_199_, p‐tau_231_, and VILIP‐1) in the analyzed subjects are presented in Table 1. The measured interassay coefficient of variability (CV) was <10%. The intraassay CV was <15% for all biomarkers used. No significant differences were found between males and females in any of the groups. There was no significant difference in the levels of CSF protein biomarkers among subjects with different *MAPT* rs242557, *MAPT* rs3785883, and *MAPT* rs7521 genotypes.

### 3.2 *MAPT* rs1467967 genotype

**Figure 1:**
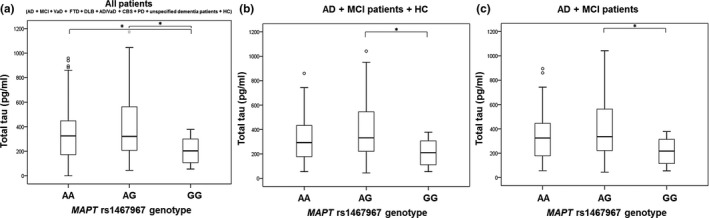
Levels of t‐tau in (a) all patients, (b) AD, MCI patients and HC, and (c) AD and MCI patients with *MAPT* rs1467967 genotype. Boxes represent the median, the 25th and 75th percentiles, and bars indicate the range of data distribution. Circles represent outliers. *p < 0.05

**Figure 2:**
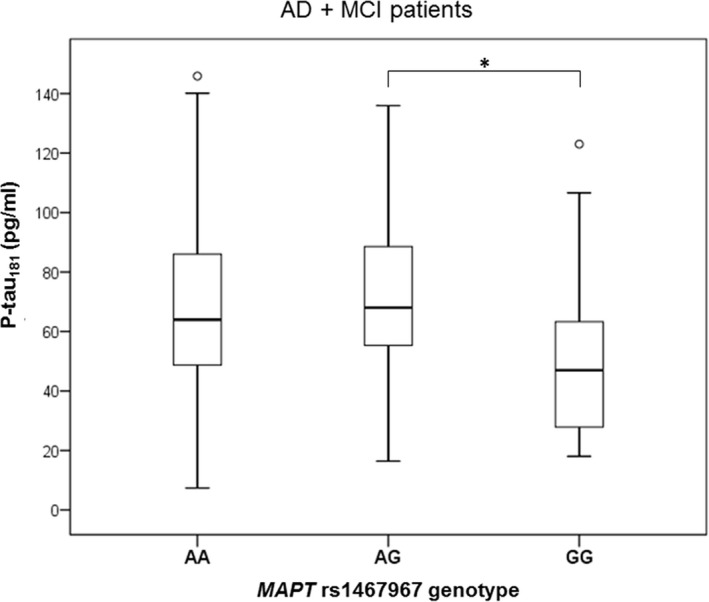
Levels of p‐tau_181_ in AD and MCI patients with *MAPT* rs1467967 genotype. Boxes represent the median, the 25th and 75th percentiles, and bars indicate the range of data distribution. Circles represent outliers. *p < 0.05

The difference in t‐tau levels was detected between patients with *MAPT* rs1467967 genotype (*H* = 11.655, *df *= 2, *p* = 0.003). T‐tau levels were significantly higher in patients with AG compared to GG *MAPT* rs1467967 genotype (*p* = 0.002) and AA compared to GG *MAPT* rs1467967 genotype (*p* = 0.022) when all patients were analyzed together (Figure 1a). The observation of increased t‐tau levels in patients with AG compared to GG *MAPT* rs1467967 genotype was confirmed when combining AD and MCI patients, and healthy controls (*p* = 0.004; Figure 1b) and in AD and MCI patients (*p* = 0.005; Figure 1c). The difference in p‐tau_181_ levels was detected between patients with *MAPT* rs1467967 genotype (*H* = 6.955, *df *= 2, *p* = 0.031). P‐tau_181_ levels were significantly higher in patients with AG in comparison to GG *MAPT* rs1467967 genotype when combining AD and MCI patients (*p* = 0.025; Figure 2). There was no significant difference in levels of Aβ_1–42_, p‐tau_199_, p‐tau_231_, and VILIP‐1 among subjects with different *MAPT* rs1467967 genotype.

### 3.3 *MAPT* rs2471738 genotype

**Figure 3:**
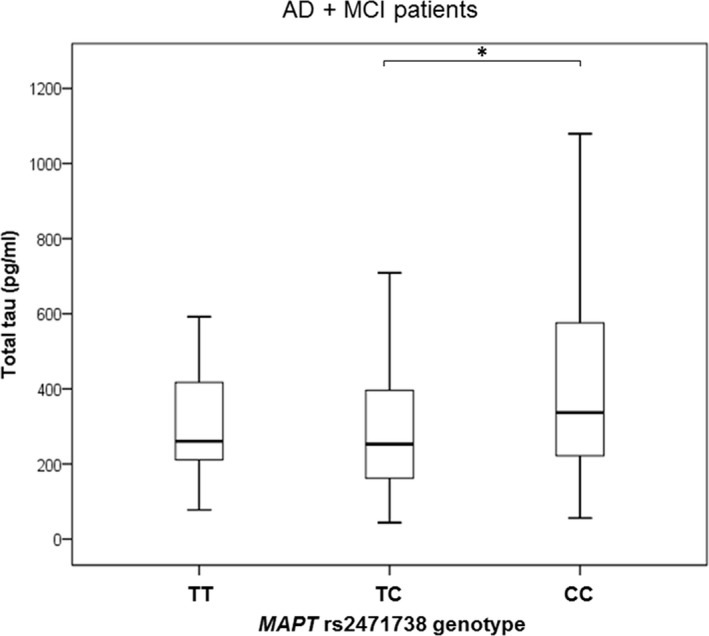
Levels of t‐tau in AD and MCI patients with *MAPT* rs2471738 genotype. Boxes represent the median, the 25th and 75th percentiles, and bars indicate the range of data distribution. Circles represent outliers. *p < 0.05

**Figure 4:**
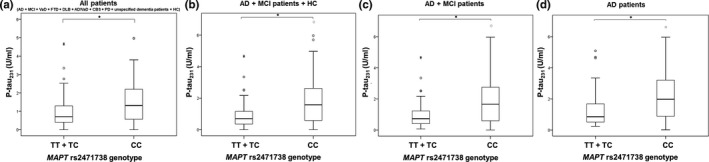
Levels of p‐tau_231_ in (a) all patients, (b) AD, MCI patients and HC, (c) AD and MCI patients, and (d) AD patients with the *MAPT* rs2471738 genotype. Boxes represent the median, the 25th and 75th percentiles, and bars indicate the range of data distribution. Circles represent outliers, and asterisks represent extreme data points. *p < 0.05

**Figure 5:**
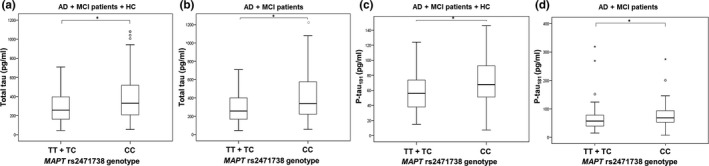
Levels of t‐tau (a, b) and p‐tau_181_ (c, d) in AD, MCI patients and HC and in AD and MCI patients with the *MAPT* rs2471738 genotype. Boxes represent the median, the 25th and 75th percentiles, and bars indicate the range of data distribution. Circles represent outliers, and asterisks represent extreme data points. *p < 0.05

The difference in t‐tau levels was detected between patients with *MAPT* rs2471738 genotype (*H* = 8.042, *df *= 2, *p* = 0.018). More precisely, t‐tau levels were significantly higher in subjects with CC in comparison to TC *MAPT* rs2471738 genotype (in AD and MCI patients; *p* = 0.017; Figure 3). Levels of p‐tau_231_ were significantly higher in subjects with CC compared to TT + TC *MAPT* rs2471738 genotype (in all patients grouped together; *U* = 3,206, *Z* = −3.621, *p* < 0.001; Figure 4a), in the combined group of AD, MCI patients and healthy controls (*U* = 1613.5, *Z* = −3.993, *p* < 0.001; Figure 4b), in AD and MCI patients (*U* = 1,467, *Z* = −3.794, *p* < 0.001; Figure 4c) and in AD patients (*U* = 671, *Z* = −3.000, *p* = 0.003; Figure 4d). T‐tau levels were significantly higher in subjects with CC in comparison to TT + TC *MAPT* rs2471738 genotype (combining AD, MCI patients and healthy controls; *U* = 2,462.5, *Z* = −2.497, *p* = 0.013; Figure 5a), and in AD and MCI patients (*U* = 2,148, *Z* = −2.823, *p* = 0.005; Figure 5b). P‐tau_181_ levels were significantly higher in subjects with CC in comparison to TT + TC *MAPT* rs2471738 genotype (combining AD, MCI patients and healthy controls; *U* = 2,485, *Z* = −2.689, *p* = 0.007; Figure 5c), and in AD and MCI patients (*U* = 2,411, *Z* = −2.534, *p* = 0.011; Figure 5d). There was no significant difference in levels of Aβ_1–42_, p‐tau_199_, and VILIP‐1 among subjects with different *MAPT* rs2471738 genotype.

### 3.4 *MAPT* haplotypes

**Figure 6:**
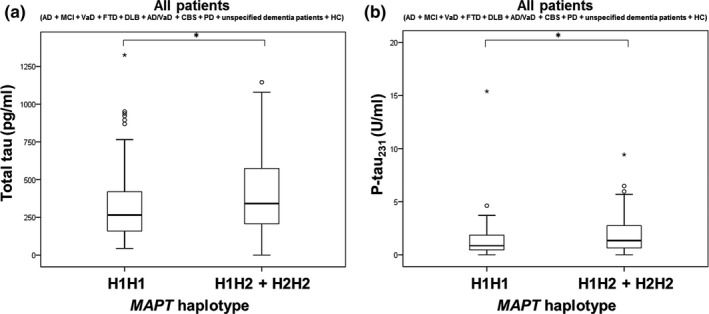
Levels of (a) t‐tau and (b) p‐tau_231_ in all patients with H1H1 and H1H2 + H2H2 *MAPT* haplotypes. Boxes represent the median, the 25th and 75th percentiles, and bars indicate the range of data distribution. Circles represent outliers, and asterisks represent extreme data points. *p < 0.05

In this Croatian cohort, 23 H1 subhaplotypes and 5 H2 subhaplotypes were detected (Table 2). T‐tau (*U* = 3,791, *Z* = −2.524, *p* = 0.012; Figure 6a) and p‐tau_231_ (*U* = 3,124.5, *Z* = −2.710, *p* = 0.007; Figure 6b) levels were significantly higher in subjects with H1H2 + H2H2 haplotype compared to H1H1 haplotype when all patients were analyzed together.

## 4 DISCUSSION

This preliminary study of a Croatian cohort investigated whether certain variants of *MAPT* gene were associated with AD pathology as it was shown that polymorphisms in the *MAPT* gene increase the risk of tauopathies (Di Maria et al., 2010; Myers et al., 2005; Pittman et al., 2005). While a limitation of this study is a low number of HC (*n* = 9), our analysis of *MAPT* polymorphisms was conducted in all patients including the HC group (228 subjects in total), as such, the small number of HC is unlikely to have influenced the outcome of the analysis. The levels of t‐tau and p‐tau_181_ were significantly higher in patients with AG compared to GG *MAPT* rs1467967 genotype. Levels of t‐tau were significantly higher in patients with AA compared to GG *MAPT* rs1467967 genotype. Levels of t‐tau were significantly higher in patients with CC compared to TC *MAPT* rs2471738 genotype. Additionally, levels of t‐tau, p‐tau_181_ and p‐tau_231_ were significantly higher in patients with CC compared to patients with TT or TC *MAPT* rs2471738 genotypes. Also, levels of t‐tau and p‐tau_231_ were significantly higher in patients with H1H2 or H2H2 haplotypes compared to patients with the H1H1 haplotype.

Previous studies reported significant differences in CSF tau levels in carriers of risk alleles in *MAPT* rs242557 (Compta et al., 2011; Laws et al., 2007), rs16940758, rs3785883, rs243511, and rs2471738 polymorphisms (Kauwe et al., 2008). Additionally, Chen et al. (2017) found an association of the A‐allele in *MAPT* rs242557 polymorphism with increased levels of t‐tau in plasma. Compta and collaborators found that carriers of the A‐allele in *MAPT* rs242557 polymorphism had increases in CSF t‐tau and p‐tau_181_ levels (Compta et al., 2011). This was observed in patients with PD, but only in those with dementia and pathological Aβ_1–42_ levels (lower than 500 pg/ml). Although Compta et al. compared the levels of t‐tau and p‐tau_181_ in PD patients with different *MAPT* rs1880753, rs1880756, rs1800547, rs1467067, rs242557, rs2471738, and rs7521 genotypes, they found no significant differences in t‐tau and p‐tau_181_ levels in contrast to the present study that reveals t‐tau and p‐tau levels to be altered in patients with different *MAPT* rs1467967 and rs2471738 genotypes. Elias‐Sonnenschein et al. (2013) tested if different *MAPT* rs1467967 and rs7521 genotypes affected the levels of Aβ_1–42_, t‐tau and p‐tau_181_ in AD patients. No significant difference in the levels of CSF biomarkers between these patients was found. However, *MAPT* rs2435211 and rs16940758 polymorphisms were related to increased t‐tau and p‐tau, respectively. In the study of Kauwe et al. (2008) in which 21 SNPs in *MAPT* gene were genotyped, an association of rs16940758, rs3785883, rs243511, and rs2471738 with p‐tau_181_ was observed. Additionally, these polymorphisms demonstrated an association with t‐tau and p‐tau_181_ in patients with pathological Aβ_1–42_ levels. This study supports our finding that t‐tau, p‐tau_181_, and p‐tau_231_ levels are significantly different in patients with different *MAPT* rs2471738 genotypes. However, in contrast to our results, the association of *MAPT* rs1467967 genotype with t‐tau and p‐tau_181_ that was also tested in that study was not observed (Kauwe et al., 2008). Although Laws et al. (2007) analyzed the association of CSF t‐tau with all polymorphisms included in our study, only an association of *MAPT* rs242557 genotype with t‐tau levels was observed. Laws et al. proposed that association between CSF tau levels and *MAPT* polymorphisms (or haplotypes) could occur through *MAPT* expression. In other words, individuals carrying risk *MAPT* alleles have a higher *MAPT* brain expression and consequently an increased neurodegeneration and leakage of tau protein in CSF (Laws et al., 2007).

The study of Ning et al. (2011) showed that *MAPT* rs1467967 polymorphism could serve as a genetic biomarker for VaD, since the rs1467967 genotypes differed between VaD patients and HC. Also, the *MAPT* rs2471738 polymorphism was associated with an increased risk for AD (Vázquez‐Higuera et al., 2009). While the meta‐analysis of Yuan, Du, Ge, Wang, & Xia (2018) showed that none of the polymorphisms analyzed in the present study (rs1467967, rs3785883, rs2471738, and rs7521), except for rs242557 that showed an association with AD, the meta‐analysis of Zhou and Wang (2017) demonstrated an association of rs242557 and rs2471738 polymorphisms (but not rs3785883 or rs1467967 polymorphisms) with AD.

The association of the H1U and H1H haplotypes and H1C haplotype with t‐tau levels was previously demonstrated (Kauwe et al., 2008; Laws et al., 2007). The H1C haplotype was shown to be a risk factor for progressive supranuclear palsy and CBS (Pittman et al., 2005), late‐onset AD (LOAD; Myers et al., 2005), and MCI (Di Maria et al., 2010), while the H2 haplotype was associated with a reduced risk for LOAD (Allen et al., 2014; Zhang et al., 2017). Our results do not support observations that individuals with the H1 haplotype have increased CSF t‐tau levels (Kauwe et al., 2008; Laws et al., 2007) and increased risk for AD or other tauopathies (Di Maria et al., 2010; Myers et al., 2005; Pittman et al., 2005), as our patients with the H2 haplotype had pathological CSF t‐tau and p‐tau levels. However, several studies failed to detect the association of the H1 haplotype with an increased risk for AD (Abraham et al., 2009; Mukherjee, Kauwe, Mayo, Morris, & Goate, 2007; Russ et al., 2001). Additionally, in the study of Min et al. (2014), patients with FTD and *MAPT* H1 haplotype had an increase in CSF p‐tau_181_ levels, while there was no difference in the levels of t‐tau. Another study found no association between *MAPT* haplotypes and CSF t‐tau, p‐tau_181_ or Aβ_1–42_ levels (Johansson, Zetterberg, Håkansson, Nissbrandt, & Blennow, 2005).

In conclusion, the present study resulted in several notable findings. The association of *MAPT* rs1467967 polymorphism with AD pathology measured by levels of CSF biomarkers was demonstrated, with CSF t‐tau and p‐tau_181_ levels being significantly higher in patients with AG compared to GG *MAPT* rs1467967 genotype and t‐tau levels being significantly higher in patients with AA compared to GG *MAPT* rs1467967 genotype. Additionally, we detected an association of the *MAPT* rs2471738 polymorphism with AD pathology that was also observed in previous studies (Kauwe et al., 2008; Vázquez‐Higuera et al., 2009). However, the *MAPT* rs2471738 risk allele detected in our study (C‐allele) differs from the risk allele detected in the study of Myers et al. (2005) and Vázquez‐Higuera et al. (2009) (T‐allele), warranting further investigation. We also observed an increase in CSF t‐tau and p‐tau levels in patients with H1H2 or H2H2 haplotypes. This finding differs from studies in which the H1 haplotype was detected as a risk haplotype for AD and other tauopathies (Di Maria et al., 2010; Myers et al., 2005), and this issue also will require additional research. We used potentially novel CSF biomarkers of AD as endophenotypes (p‐tau_199_, p‐tau_231_, and VILIP‐1), while previous studies testing the association of *MAPT* polymorphisms with AD used only core CSF biomarkers as endophenotypes (Aβ_1–42_, t‐tau, p‐tau_181_). The p‐tau_231_ endophenotype showed significant difference between groups of patients with different *MAPT* genotypes. Finally, we detected 23 H1 and 5 H2 *MAPT* subhaplotypes in this Croatian cohort, revealing that *MAPT* haplotype‐tagging polymorphisms and *MAPT* haplotypes should be further tested as potential genetic biomarkers of AD.

## DATA AVAILABILITY

The datasets used and analyzed during the current study are available from the corresponding author on reasonable request.

## CONFLICT OF INTEREST

None declared.

## AUTHOR CONTRIBUTIONS

GŠ conceived and directed the study. NK and FB performed the clinical assessments and lumbar puncture. NW and RdS determined *MAPT* haplotypes. MNP and NP determined *MAPT* genotypes. MBL and GŠ determined levels of CSF biomarkers. MBL, ZS and GŠ completed statistical analysis. PRH substantially contributed to the interpretation of data and to manuscript preparation. All authors contributed to revising and editing the manuscript critically for important intellectual content. All authors read and approved the final version of the manuscript. All authors met the criteria for authorship, as defined by the International Committee of Medical Journal Editors.
